# Concomitant septic arthritis and osteomyelitis of the hip in young children; a new pathophysiological hypothesis suggested by MRI enhancement pattern

**DOI:** 10.1186/s12880-015-0057-0

**Published:** 2015-05-19

**Authors:** Laura Merlini, Mehrak Anooshiravani, Dimitri Ceroni

**Affiliations:** Unit of Pediatric Radiology Geneva University Hospital HUG, 6 Willy-Donzé, Geneva, 1205 Switzerland; Unit of Pediatric Orthopedics, University Hospital, Geneva, Switzerland

## Abstract

**Background:**

In children, septic arthritis (SA) of the hip is either primary or concomitant with acute haematogenous osteomyelitis (AHO). However, seldom, patients with isolated SA at presentation, may later show osteomyelitis in the metaphysis. The aim of this study was to elaborate a physiopathological hypothesis based on the peculiar MRI findings to explain the onset of AHO after SA.

**Methods:**

Cases of acute infection of the hip admitted between January 2010 and December 2013 were retrospectively reviewed to assess radiographic and MRI features, as well as bacteriological findings. Only children with isolated SA were included in this study, whereas cases of concomitant SA and AHO at presentation were excluded.

**Results:**

Ten patients met the inclusion criteria. Six (1–11 months) demonstrated, on the initial MRI, decreased perfusion on gadolinium enhanced fat-suppressed T1-weighted sequence of the femoral epiphysis and developed one month later metaphyseal AHO. Four (5–14 years) did not show decreased perfusion and did not develop AHO on follow-up. The type of germ involved influenced neither the type of enhancement pattern nor the outcome.

**Conclusions:**

Age under one year and decreased perfusion of the affected femoral epiphysis increases the risk of secondary AHO. Our study is the first report in human medicine supporting the physiopathological hypothesis described by Alderson et al. in an animal model: primary infection can originally affect the joint, then penetrate the epiphyseal cartilage, and finally spread into the metaphyseal region through transphyseal vessels present only in the first 12/18 months of life.

## Background

Early diagnosis of hip septic arthritis (SA) is paramount to prevent immediate joint damage and to avoid late devastating complications in infants, especially in newborns. [1–5]. Children with concomitant SA and AHO have statistically less favorable outcome, and require both more surgical procedures and longer antibiotic therapy than patients with isolated SA or AHO [6–8].

In the literature, SA is reported as a frequent complication of AHO in children [9–11] and not the other way round. According to the classical pathogenetic theory, bone sepsis in the early stages of osteomyelitis typically arises in the metaphyseal side of the growth plate of long tubular bones favoured by a peculiar aspect of vessels beneath the physeal plate: in fact they present a dilated turning point with low velocity flow allowing bacterial development [12]. In addition, the endothelium wall of the metaphyseal capillaries has gaps that allow the passage of bacteria and, moreover, phagocytic cells are absent in this region of the bone [13]. Translocated pathogens then find locally favourable conditions to proliferate due to the absence of phagocytic cells in this region of the bone. Furthermore, infection easily spreads to the articular cavity due to the low attachment of capsular structures in young children, extensively involving the metaphyseal region [12, 14].

In recent years, MRI has gained interest to establish the diagnosis of osteoarticular infection of the hip, especially for excluding concomitant SA with AHO [15–18].

However, in few situations, the classical MRI signs of AHO are lacking at presentation and bone infection manifests later. This study attempts to suggest a physiopathological hypothesis to explain the onset of AHO after SA and to identify early MRI signs predicting which patients are at risk secondary AHO.

## Methods

After approval by the Institutional Review Board (University Hospital of Geneva 09-029R, Mat-Ped 09-008R), medical charts of all children admitted to our tertiary care hospital from January 2010 to December 2013 for suspicion of osteoarticular infection of the hip were retrospectively reviewed. Analyses were based on clinical records, demographics (age and gender), body temperature, and laboratory data including bacterial cultures (from blood and bone samples), white blood cell count (WBC), platelet count, erythrocyte sedimentation rate (ESR) and serum C-reactive protein (CRP). Since 2007, all samples are also analyzed using either a broad range PCR assay or a new, real-time PCR assay specific to *K. kingae*. Radiological studies of the hip were available for all patients, and included plain radiographs, and MRIs.

For children less than 5 years, MRI was performed under general anesthesia whereas the examination was realized without sedation in older children. MRI studies were performed on a 1.5-T Avanto (Siemens) machine and the protocol included coronal turbo spin echo T1-weighted sequences (TR/TE = 500/ 12), axial turbo spin echo T2-weighted images (TR/ TE =6420/99), 3 days T2 STIR SPACE images (TR/ TE =2000/201, thickness = 1,3–1,5 mm, isometric), and coronal (TR/TE = 639/13) and axial (TR/TE = 350/8,8) fat-suppressed spin-echo T1-weighted images after intravenous administration of gadopentetate dimeglumine (Dotarem) at a dose of 0.1 mmol/kg body weight. Contrast enhanced images were obtained directly after contrast injection and no dynamic sequences were obtained. Then children were transferred under general anesthesia to the operating room where joint fluid or bone aspirate specimen were taken and sent to the laboratory for Gram staining and immediate inoculation onto Columbia blood agar (incubated under anaerobic conditions), CDC anaerobe 5 % sheep blood agar (incubated under anaerobic conditions), chocolate agar (incubated in a CO_2_-enriched atmosphere), and brain-heart broth. Incubation time was 10 days. All samples were also analyzed using either a broad range PCR assay or a new, real-time PCR assay specific to *K. kingae*.

MRI studies were anonymized, coded and transferred to a dedicated computer station and stored in random order. Two senior pediatric radiologists (LM and MA) independently reviewed MR images. Only patients with MR signs of isolated SA were included in this study, whereas children with concomitant SA and AHO at initial investigation were excluded from the study. According to the literature, we analyzed the images as follows:

1. AHO was diagnosed when there was focally decreased marrow signal intensity on T1-weighted images and focally increased marrow signal intensity on fluid-sensitive images (fat-suppressed T2-Weighted and STIR sequences). On enhanced, fat- suppressed T1-weighted images, osteomyelitis usually presents as focal abnormal bone marrow enhancement [17, 19] and sometimes, in children, as focal enhancement defects of the epiphyseal cartilage [20].
2. We considered isolated SA when there was joint effusion with or without soft tissue involvement. Joint effusion was quantified as mild if there was only a small hyperintense line on T2-Weighted sequences, moderate if there was a more significant effusion and important if the femoral head was eccentric.
3. The enhancement pattern of the femoral epiphyseal head cartilage and bone nucleus was qualitatively compared to the contralateral side on substraction images, and on slices with the most intense enhancement.

**Fig. 1:**
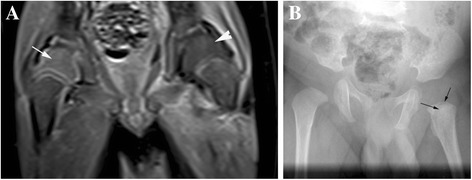
**a** and **b** - 1 month-old boy. **a**) coronal contrast-enhanced fat-suppressed T1W: on the left femoral epiphysis hypointensity (arrowhead) in comparison to a homogeneously enhanced right femoral epiphysis (arrow). Epiphyseal nuclei are not yet present. **b**) X Ray study performed one month later showing a small lytic lesion (arrows) on the left side

**Fig. 2:**
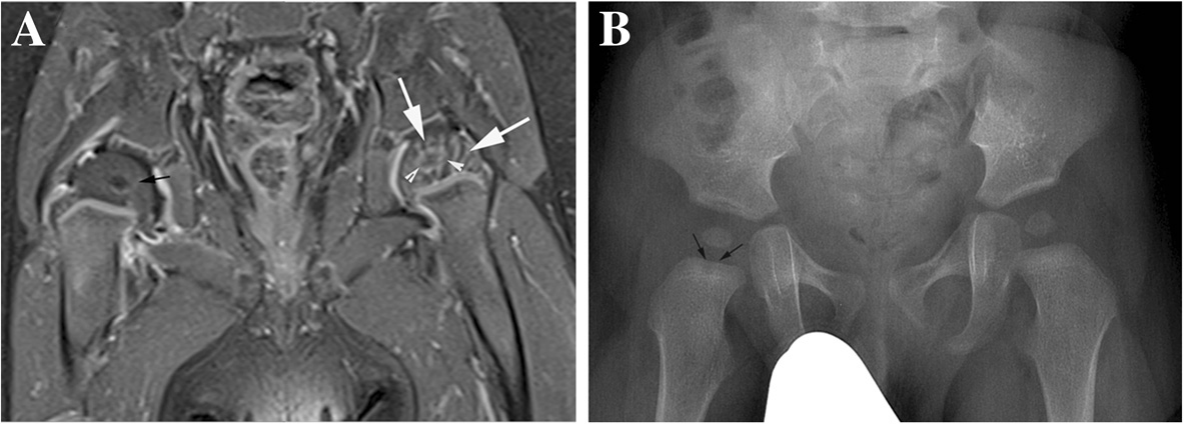
**a** and **b** - 10 month-old boy **a**) coronal contrast-enhanced fat-suppressed T1W : femoral epiphyseal nuclei are present. The pathological right femoral cartilage lacks the « striate » normal aspect (white arrows on the left side), the nucleus is uniformly hypointense (red arrow on the right side) in comparison with the normal side (arrowheads) and the surrounding ring does not enhance. Metaphyseal right region shows no abnormalities. **b**) X Ray study performed one month later. A small lytic lesion is visible on the metaphyseal side of physis (arrows) on the right side

On the Fat SAT T1 enhanced sequence, the normal cartilaginous femoral epiphysis appears uniformly hyperintense (Fig. 1a arrow) before the appearance of femoral head ossification centers. With the appearance of ossification centers, normal cartilaginous femoral epiphysis presents a « striate » aspect due to enhanced vessels (Fig. 2a arrows). Ossification centers are uniformly enhanced in comparison with the pre-contrast appearance and surrounded by a thin hyperintense line (Fig. 2a arrowhead). The cartilaginous part reduces with age and this « striate » aspect usually disappears by the second year of life.

**Fig. 3:**
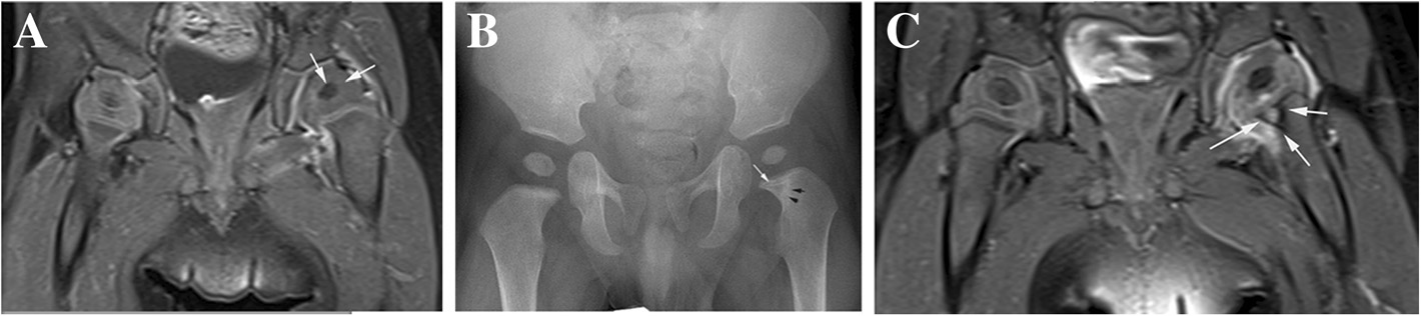
**a**, **b** and **c** - 6 month-old boy **a**) Contrast-enhanced fat-suppressed spin-echo T1- weighted coronal image shows hypoperfusion of the left femoral epiphysis. **b**) X Ray study performed one month later showed a small lytic area on the metaphyseal side of the physis (white arrow) with a sclerotic halo (black arrows): the epiphyseal nucleus was also smaller on the pathological side (left). **c**) Contrast-enhanced fat-suppressed spin-echo T1-weighted coronal sequences performed one month later showing metaphyseal bone lesion on the left side. The left femoral nucleus showed hypointensity and was smaller in comparison to the right side

In our study, decreased perfusion was considered when the pathological femoral epiphysis was uniformly hypointense in comparison to the contralateral side (Fig. 1a arrowhead) if the ossification centers were not present. If present, when they were hypointense, the surrounding line did not enhance and the “striate” aspect of the cartilaginous part was absent (Fig. 3a arrows). We did not measure enhancement ratios.

Conventional X ray studies of the hip were performed in all cases one month after the MRI and images were reviewed by the same two radiologists: signs of AHO sequelae (lytic lesions, sclerosis, or disappearing of ossification centers) were reported if present.

## Results

From January 2010 to December 2013, 22 patients were investigated with MRI in our tertiary care hospital for suspicion of osteoarticular acute infection of the hip. Twelve MRIs initially demonstrated either concomitant SA and AHO or isolated AHO; these cases were excluded of the present study. In 10 cases (45 % of all cases) MRI showed isolated SA without MRI signs of AHO at presentation and these patients were included in the study. The duration of symptoms ranged between 2 and 7 days at the time of the MRI . Body temperature (fever) was between 38.5 and 39.2 °C for 8 patients whereas 2 patients had 37 and 37.6 °C*.* CRP measurements also ranged between 12 and 180 mg/L (normal range 0–5 mg/L) and erythrocyte sedimentation rate test (ESR) between 19 and 100 mm/h and leucocytes between 9300 and 23.000 cells/μL. Clinical and biological inflammatory responses to *K.kingae* infection were mild-to-moderate in comparison with cases of Gram-positive bacilla infections.

Four patients (3 girls and one boy, mean age 7 years, range 5–14 years) had joint effusion of the hip and did not show decreased perfusion MRI pattern of the femoral epiphysis on fat-suppressed gadolinium-enhanced coronal T1-weighted MRI. The joint fluid effusion was mild in all four cases. The causative organism was *Streptococcus agalactae* in one case, *Streptococcus pneumonia* in two others and was unknown in the fourth. None of the children without this enhancement pattern (4 out of 10) later developed osteomyelitis sequela. Six patients (4 boys and 2 girls, mean 6.3 months, range 1–11 months) presented joint effusion and decreased perfusion of the femoral epiphysis on fat-suppressed gadolinium-enhanced coronal T1-weighted MRI (Fig. 1a, Fig. 2a, and Fig. 3a). In two patients, the causative organism was *Kingella kingae,* in three Gram Positive germs (one *Streptococcus pneumoniae*, one *Streptococcus agalactae* and one *Staphylococcus aureus*) and in one patient bacterial investigation was negative. The joint fluid effusion was mild in five cases and moderate in one. All patients (6 out of 10) with decreased enhancement of gadolinium in the affected hip compared to the contralateral hip, developed metaphyseal small lytic lesions on follow-up (one month later) X rays (Fig. 1b, Fig. 2b and Fig. 3b arrows) and in one case the ossification center disappeared. All of them were younger than one year. The type of germ involved influenced neither the type of enhancement pattern nor the outcome.

In one case with decreased enhancement, a second MRI performed one month later, clearly showed morphological and signal abnormalities of the growth plate and a round abscess in the metaphysis (Fig. 3c): decreased enhancement of the femoral cartilaginous head was no more visible but there was persistent relative hypointensity of the femoral nucleus which was also smaller in comparison to the contralateral side.

## Discussion

The findings of the present study suggest that patients with isolated SA at presentation may later show bone lesions in the metaphysis suggestive of secondary osteomyelitis. A direct correlation seems to exist between a decreased perfusion of the femoral epiphysis on MRI in children below the age of 12 months and the later onset of osseous lesions in the metaphysis consistent with AHO on follow-up. In our experience, the type of germ involved influences neither the enhancement pattern nor the outcome. According to the recent literature, decreased perfusion of the femoral epiphysis on enhanced fat- suppressed T1-weighted MRI has already been described, but it was until now considered as a usual finding of SA [9–11], and in no case interpreted as a sign suggestive of a bone infection of the epiphysis. One study reported that epiphyseal cartilage enhancement defects may occur in the setting of AHO, but its significance was not investigated and remained uncertain. [20] In fact, SA is classically reported as a very common complication of AHO in little children due to the peculiar anatomy of the immature hip and the pathophysiology of pediatric AHO.

The cases reported in our study demonstrate that SA clearly preceded metaphyseal bony infectious lesions and not the opposite. In the literature, SA is classically reported as a frequent complication of AHO and not the other way round [12] as in our findings. We have thus considered two hypothesis in order to explain our findings. The first is that, at presentation, AHO had already started in the metaphysis but MRI was not yet able to visualize the concomitant bone lesion. This explanation would be in agreement with the classical pathophysiological theory of AHO in children [12, 14], where infection typically arises in the metaphysis of long tubular bones due to its rich vascular supply in young children. However, it would be difficult to explain why MRI did not detect any initial signs of metaphyseal bone infection. The second and more rational hypothesis can be formulated on the basis of the observations of Alderson et al. [20]: they investigated concurrent acute childhood osteomyelitis and septic arthritis using avian models which closely resemble human disease. They observed that, after intra-articular injection, bacteria penetrated the deeper epiphyseal cartilage and within 24 h occluded blood vessels. Furthermore, once bacteria were established in the epiphysis, spread occurred across the growth plate into the metaphyseal region due to the presence of transphyseal vessels [12, 14]. This vascular pattern seems to be specific of children in the first 12 to 18 months of life and we can assume, as suggested by Alderson and al, that these vessels may allow the translocation of bacteria in both directions from or to the epiphysis. According to this theory, the explanation of reduced enhancement of the femoral epiphysis and the lack of the normal “striate” aspect is the early thrombosis of epiphyseal cartilage vessels.

According to this theory, it would be more likely that AHO develops first in the epiphysis and then in the metaphysis. In our cases, however, no signal anomalies were detected in the epiphysis on the initial basic T1 and T2 sequences. The reason why may be that, unlike metaphyseal vessels, epiphyseal vessels have a linear morphology, providing much less favourable conditions for bacteria to proliferate.

The present study is a case-series with low-evidence level and has the limitations of being retrospective and including a small population. However we think that our findings deserve to be further investigated by prospective studies with follow-up investigations in patients with isolated SA at presentation.

## Conclusion

Infants below the age of one year, with isolated septic joint effusion and decreased perfusion of the femoral epiphysis on enhanced fat- suppressed T1-weighted MRI at presentation, will likely develop metaphyseal AHO and should be treated accordingly. In children after the age of one we did not observe this enhancement pattern, and AHO was less likely.

On the basis of our observations we suggest a new physiopathological hypothesis, already suggested in the literature on the basis of avian models which closely resemble human disease. Finally, we would recommend to perform a contrast-enhanced MR in pediatric hip infection.

## References

[1] Forlin EE, Milani CC (2008). Sequelae of septic arthritis of the hip in children: a new classification and a review of 41 hips. J Pediatr Orthop.

[2] Morrey BF, Bianco AJ, Rhodes KH (1975). Septic arthritis in children. Orthop Clin North Am.

[3] Fabry G, Meire E (1983). Septic arthritis of the hip in children: poor results after late and inadequate treatment. J Pediatr Orthop.

[4] Berberian GG, Firpo VV, Soto AA, Lopez MÒ, Julio Torroija J, Cecilia Castro C (2010). Osteoarthritis in the neonate: risk factors and outcome. Braz J Infect Dis.

[5] Rutz EE, Spoerri MM (2013). Septic arthritis of the paediatric hip - a review of current diagnostic approaches and therapeutic concepts. Acta Orthop Belg.

[6] Tetzlaff TR, McCracken GH, Nelson JD (1978). Oral antibiotic therapy for skeletal infections of children. II. Therapy of osteomyelitis and suppurative arthritis. J Pediatr.

[7] Unkila Kallio L, Kallio MJ, Peltola H (1994). The usefulness of C-reactive protein levels in the identification of concurrent septic arthritis in children who have acute hematogenous osteomyelitis. A comparison with the usefulness of the erythrocyte sedimentation rate and the white blood-cell count. J Bone Joint Surg (Am Vol).

[8] Goergens ED, McEvoy A, Watson M, Barrett IR (2005). Acute osteomyelitis and septic arthritis in children. J Paediatr Child Health.

[9] Kwack KK-S, Cho JHJ, Lee JHJ, Oh K, Ki K, Kim S (2007). Septic arthritis versus transient synovitis of the hip: gadolinium-enhanced MRI finding of decreased perfusion at the femoral epiphysis. AJR Am J Roentgenol.

[10] Yang WJW, Im SAS, Lim GG-Y, Chun H, Ho J, Jung N (2006). MR imaging of transient synovitis: differentiation from septic arthritis. Pediatr Radiol.

[11] Lee SK, Suh KJ, Kim YW, Ryeom HK, Lee JM, Chang Y (1999). Septic arthritis versus transient synovitis at MR imaging: preliminary assessment with signal intensity alterations in bone marrow. Radiology.

[12] Trueta J (1963). The 3 types of acute hematogenous osteomyelitis. Schweiz Med Wochenschr.

[13] Ceroni DD, Kampouroglou GG, Valaikaite RR, Anderson Della Llana R, Salvo D (2014). Osteoarticular infections in young children: what has changed over the last years?. Swiss Med Wkly.

[14] Ogden JA (1979). Pediatric osteomyelitis and septic arthritis: the pathology of neonatal disease. Yale J Biol Med.

[15] Saigal GG, Azouz EMG, Abdenour GG (2004). Imaging of osteomyelitis with special reference to children. Semin Musculoskelet Radiol.

[16] Averill LWL, Hernandez AA, Gonzalez LL, Pea AHA, Jaramillo D (2009). Diagnosis of osteomyelitis in children: utility of fat-suppressed contrast-enhanced MRI. AJR Am J Roentgenol.

[17] Dangman BC, Hoffer FA, Rand FF, O’Rourke EJ (1992). Osteomyelitis in children: gadolinium-enhanced MR imaging. Radiology.

[18] Montgomery COC, Siegel EE, Blasier RDR, Suva LJL (2013). Concurrent septic arthritis and osteomyelitis in children. J Pediatr Orthop.

[19] Johnson DPD, Hernanz Schulman MM, Martus JEJ, Lovejoy SA, Yu C, Kan JH (2010). Significance of epiphyseal cartilage enhancement defects in pediatric osteomyelitis identified by MRI with surgical correlation. Pediatric radiology.

[20] Alderson M, Speers D, Emslie K, Nada S (1986). Acute haematogenous osteomyelitis and septic arthritis-a single disease. An hypothesis based upon the presence of transphyseal blood vessels. J Bone Joint Surg Br Vol.

